# Usefulness of a Kampo Medicine on Stress-Induced Delayed Gastric Emptying in Mice

**DOI:** 10.1155/2020/3797219

**Published:** 2020-01-31

**Authors:** Sachiko Mogami, Ryutaro Arita, Miwa Nahata, Naoki Fujitsuka, Shin Takayama, Tadashi Ishii

**Affiliations:** ^1^Tsumura Kampo Research Laboratories, Tsumura & Co., Ibaraki, Japan; ^2^Department of Education and Support for Regional Medicine, Department of Kampo Medicine, Tohoku University Hospital, Sendai, Miyagi 980-8574, Japan; ^3^Department of Kampo and Integrative Medicine, Tohoku University Graduate School of Medicine, Sendai, Miyagi 980-8574, Japan

## Abstract

Anxiety and depression often occur with gastrointestinal symptoms. Although the Japanese traditional medicine (Kampo medicine) bukuryoingohangekobokuto (BGH) is approved for treating anxiety, neurotic gastritis, and heartburn, its effect on gastrointestinal motility remains poorly known. This study aimed to examine the effect of BGH on delayed gastric emptying in stress model mice and clarified its action mechanism. Seven-week-old C57BL/6 male mice were acclimated for a week and fasted overnight. Stress hormone, corticotropin-releasing factor (CRF), was intracerebroventricularly injected to mice, and solid nutrient meal (ground chow and distilled water) was orally administered 1 hour after. Gastric contents were collected to evaluate gastric emptying rates by measuring its dry weight. Injection of CRF (0.3 or 1.0 *μ*g/mouse) significantly delayed the 2-hour gastric emptying in mice. BGH (1.0 g/kg), which was administered 30 minutes before the CRF injection, significantly ameliorated the delayed gastric emptying induced by CRF (0.3 *μ*g/mouse). BGH (0.5, 1.0 g/kg) significantly enhanced the 1-hour gastric emptying and slightly increased the 2-hour gastric emptying in mice without CRF injection. *In vitro* functional assays showed that components of BGH antagonized or inhibited CRF type-2, dopamine D2/D3, neuropeptide Y Y2 receptors, or acetylcholinesterase. In conclusion, the components of BGH may exert synergistic effects on improving gastric emptying via various targets. BGH is considered to be potentially useful for treating gastrointestinal dysmotility with psychological symptoms.

## 1. Introduction

Although patients rarely describe their psychiatric symptoms when their major complaints include gastrointestinal disorders, psychological symptoms such as anxiety and depression may possibly lie behind gastrointestinal symptoms. Psychosocial disturbance is highly prevalent in patients with functional gastrointestinal disorder [1–4]. Moreover, elderly people who likely experience depression and gastrointestinal diseases are at the risk of facing a high total symptom burden [5], leading to a poor quality of life [6] and increased use of health services [7, 8]. Thus, appropriate management for these multiple symptoms is required.

Herbal therapies have been used in Asia and across the world for centuries, and it is currently manufactured as Japanese traditional medicines (Kampo medicines). Almost 90% of physicians in Japan use Kampo medicines in daily practice under the approval of the Japanese Ministry of Health, Labour and Welfare. Because of multitargeted, multicomponent characteristics, a Kampo medicine may have the potential to deal with multiple symptoms. Thus, they are expected to solve the problem of polypharmacy, which is a well-known risk for adverse drug reactions in the elderly patients [9]. Antianxiety/antidepressant effects are reported in various crude drug extracts or their constituent compounds, such as Magnolia Bark [10], Perilla Herb [11, 12], and ginger [13]. Hangekobokuto, which contains these crude drugs as its constituents, is one of the “Kampo medicines” and does not only exhibit antidepressive effects in mice [14, 15] but also increase the gastric emptying rate and improve gastrointestinal symptoms in patients with functional dyspepsia [16, 17], although precise mechanism is not elucidated. Bukuryoingohangekobokuto (BGH), which is approved for clinical application to patients suffering anxiety, neurotic gastritis, or heartburn, is composed of the same crude drugs as hangekobokuto but also includes *Citrus unshiu* peel, Ginseng, Immature Orange, and *Atractylodes lancea* rhizome. *Citrus unshiu* Peel–derived hesperidin [18], Immature Orange–derived meranzin hydrate [19], and *Atractylodes lancea* rhizome–derived *β*-eudesmol [20] ameliorate delayed gastric emptying. In addition, *Atractylodes lancea* rhizome–derived atractylodin [21] potentiates the receptor signaling of the prokinetic hormone ghrelin. Thus, BGH is considered to be more effective to treat gastrointestinal symptoms; however, the effects on digestive symptoms have remained unexplored.

The present study aimed to examine the effect of BGH in stress model mice with delayed gastric emptying induced by central corticotropin-releasing factor (CRF) and to clarify its action mechanism.

## 2. Materials and Methods

### 2.1. Animals

Seven-week-old male C57BL/6 mice (weighing 20–27 g) were purchased from Charles River Laboratories (Tokyo, Japan). Five mice were housed in a plastic cage (230 × 310 × 155 mm) with bedding materials and acclimated for a week in a controlled environment (20–26°C, 30–70% humidity) under a 12-hour light/dark cycle with free access to standard chow and water. This study was approved by and conducted according to the guidelines of the experimental animal ethics committees of Tsumura & Co. (Tokyo, Japan; Permit No. 14-094-1).

### 2.2. BGH Administration

BGH was manufactured by Tsumura & Co., complying with the quality standards authorized in the Japanese Pharmacopoeia, which was published under the authority of the Japanese Ministry of Health, Labour and Welfare. Briefly, BGH was obtained by spray-drying a hot water extract of a mixture of the following nine crude drugs: Pinellia Tuber (*Pinelliae tuber*), 6.0 g; Poria Sclerotium (*Poria*), 5.0 g; Atractylodes Lancea Rhizome (*Atractylodis lanceae rhizoma*), 4.0 g; Magnolia Bark (*Magnoliae cortex*), 3.0 g; Citrus Unshiu Peel (*Citri unshiu pericarpium*), 3.0 g; Ginseng (*Ginseng radix*), 3.0 g; Perilla Herb (*Perillae herba*), 2.0 g; Immature Orange (*Aurantii fructus immaturus*), 1.5 g; and ginger (*Zingiberis rhizoma*), 1.0 g. BGH was suspended in distilled water and orally administered to mice. Distilled water was administered as vehicle. BGH dose at 1.0 g/kg is approximately equivalent to a typical clinical dose when converted based on the body surface area, which is recommended by the Food and Drug Administration.

### 2.3. CRF Administration

CRF (4136-s; Peptide Institute, Inc., Osaka, Japan) was dissolved in 0.1% acetic acid/saline (which was also used as vehicle) and administered to mice via intracerebroventricular (ICV) injection under short isoflurane inhalation anesthesia using RC2 Rodent Circuit Controller (VetEquip Inc., Livermore, CA, USA). Direct ICV administration was performed according to a previous report [22] by a skilled individual who have confirmed the location of ICV injection through dye infusions with a success rate of 97%. A 26-gage stainless-steel needle that was attached to PE-10 tubing fitted to a 10 *μ*L microsyringe was inserted into the brain (2.6 mm below the skull surface, and 1.0 mm lateral and 0.5 mm anterior to the bregma) of lightly held mice. Then, 5 *μ*L of CRF was injected over 30 seconds. After the injection, mice were removed from anesthesia and became fully conscious within few minutes. Mice that showed visible bleeding or abnormal behaviors following ICV administration were excluded from the analysis because of possible inadequacy of the administration procedure.

### 2.4. Evaluation of Gastric Emptying of Solid Food

Solid gastric emptying was evaluated using a test meal containing 20 g of ground meal and 40.61 mL of distilled water. The mice were orally administered with the test meal (0.2 mL). After 1 or 2 hours, mice were anesthetized by isoflurane inhalation, and cardiac and pyloric sphincters were clamped by forceps after the laparotomy. Mice were then sacrificed by exsanguination by transecting the abdominal aorta, and the inferior vena cava and stomach were excised from the abdominal cavity. The gastric contents were collected from the stomach, dried, and then weighed. Similarly, gastric contents were collected from 0% control mice (*n* = 5) immediately after the test meal administration to recover the entire dose of the test meal. The gastric emptying of solid meal was calculated using the equation below:

(1) $$\begin{array}{c} \text{Gastric emptying}\left(\%\right)=100\times \left(1-\frac{\text{weight of gastric contents}}{\text{average weight of gastric contents from }0\%\text{ control mice}}\right). \end{array}$$

Mice with error in administration or with gastric contents full of foreign materials (pelage, bedding materials, etc.) that cannot be totally eliminated were removed from the calculations.

### 2.5. Animal Experiments

**Figure 1:**
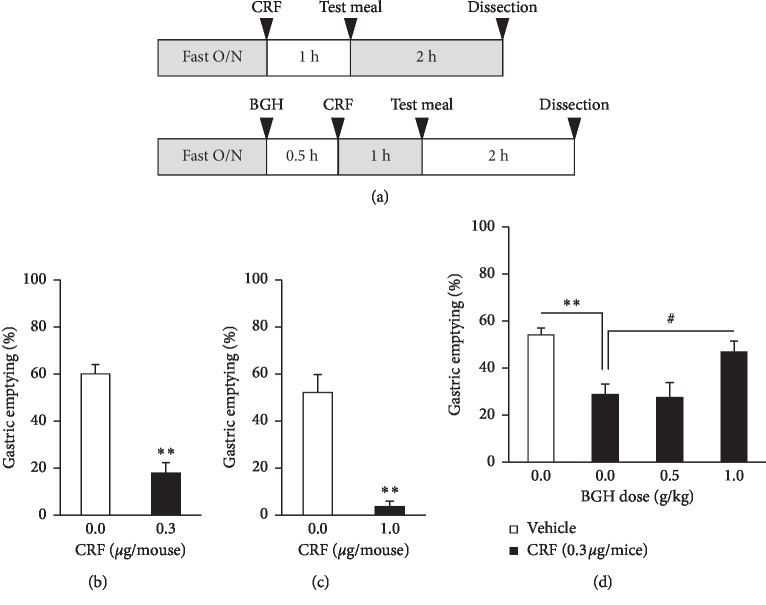
(a) Experimental protocol. (b, c) Gastric emptying rates after corticotropin-releasing factor (CRF) intracerebroventricular (ICV) administration at 0.3 *μ*g/mouse (b; *n* = 7 in each group) and 1.0 *μ*g/mouse (c; *n* = 7 in vehicle-treated and *n* = 8 in CRF-treated group). (d) Gastric emptying rates after the administration of CRF (0.3 *μ*g/mouse, ICV) and bukuryoingohangekobokuto (BGH; 0.5 and 1.0 g/kg, orally); *n* = 18 in vehicle-treated group, *n* = 16 in CRF group, *n* = 14 in CRF/BGH (0.5 g/kg) group, and *n* = 18 in CRF/BGH (1.0 g/kg) group. Data are means ± SE. ^*∗∗*^*P* < 0.01 by Student's *t*-test or Aspin−Welch *t*-test. ^#^*P* < 0.05 by Dunnett test.

Experiment 1: Mice were grouped randomly according to body weight into 2 groups (vehicle- and CRF-treated, *n* = 10/group) or 4 groups (vehicle, CRF, CRF/BGH (0.5 g/kg), and CRF/BGH (1.0 g/kg), *n* = 20/group). After overnight food deprivation in the home cage, mice were administered with CRF (0.3 or 1.0 *μ*g/mouse) via ICV injection and test meal orally administered 1 hour after. Then, 2 hours after test meal administration, gastric content was collected (13 : 00–15 : 00) to evaluate solid gastric emptying. BGH (0.5, 1.0 g/kg) was orally administered to mice, 30 minutes before CRF (0.3 *μ*g/mouse) ICV administration (Figure 1(a)).

**Figure 2:**
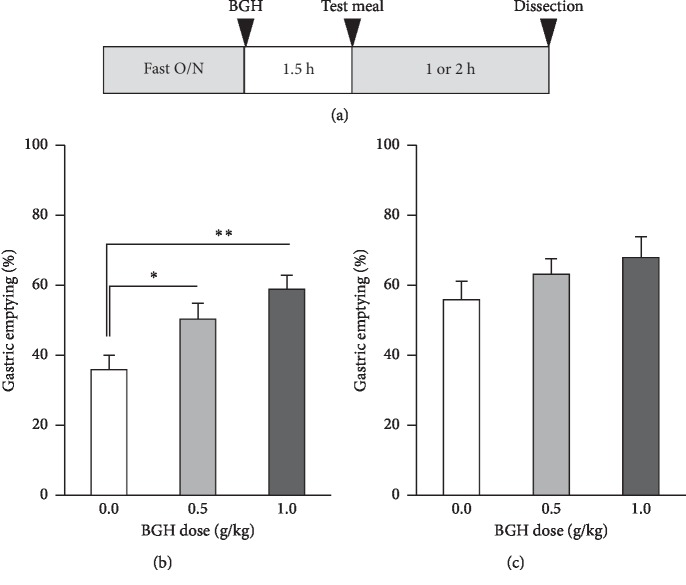
(a) Experimental protocol. (b, c) Effect of bukuryoingohangekobokuto (BGH; 0.5 and 1.0 g/kg, orally) on 1 hour (b; *n* = 10 in BGH 0 and 0.5 g/kg group and *n* = 9 in BGH 1.0 g/kg group) and 2 hour (c; *n* = 10 in each group) gastric emptying rates. Data are means ± SE. ^*∗*^, ^*∗∗*^*P* < 0.05, 0.01 by Dunnett test, respectively.

Experiment 2: Mice were grouped randomly according to body weight into 3 groups (BGH: 0, 0.5, 1 g/kg, *n* = 10/group). After overnight food deprivation in the home cage, mice were administered with BGH orally, followed by test meal orally 1.5 hour after. Then, 1 or 2 hours after test meal administration, gastric content was collected (13 : 00–15 : 00) to evaluate solid gastric emptying (Figure 2(a)).

### 2.6. *In Vitro* Functional Assays to Assess Receptor and Enzyme Antagonistic Activities of BGH Components

Assays were performed at Eurofins Discovery (MO, USA). Briefly, evaluation of the inhibitory activities against human recombinant acetylcholinesterase (AChE) expressed in HEK-293 cells was performed by preincubation with test compound and/or vehicle with 41ng/ml of enzyme for 15 minutes at 25°C in phosphate buffer pH 7.4. After 20 minute incubation with 0.7 mM acetylthiocholine iodide and 0.5 mM 5,5-dithiobis-2-nitrobenzoic acid, the spectrophotometric absorbance was read at 405 nm (Catalog No. 104010). Evaluation of the antagonistic activities against human CRF2 receptor transfected in HEK-293 cells was performed by measuring the effects of the test compounds on cAMP production induced by 100 nM human CRF incubated for 30 minutes at room temperature via the HTRF detection method (Catalog No. 2086). Evaluation of the antagonistic activities against human dopamine D2, D3, and neuropeptide Y (NPY) Y2 receptors expressed in CHO-K1 cells, CHO-K1, and KAN-TS cells, respectively, was performed by preincubation of the test compounds and/or vehicle with the membrane proteins (0.1, 0.04, 0.04 mg/ml, respectively) and GDP (10, 1, 0.001 mM) in 20 mM HEPES, pH 7.4, 100 mM NaCl, 10 mM MgCl_2_, 1 mM DTT, and 1 mM EDTA for 20 minutes at 25°C, and SPA beads are then added for another 60 minutes at 30°C. The reaction was initiated by 0.3 nM [^35^S]GTP*γ*S for an additional incubation period (15, 30, 30 minute). Inhibition on increase of [^35^S]GTPS binding response induced by dopamine (10, 0.03 mM) or 3 nM human NPY was assessed as receptor antagonist activities of test compounds (Catalog No. 310200, 310400, 332520). Evaluation of the antagonistic activities of shogaol and gingerol against human dopamine NPY Y2 receptor expressed in RBL cells was performed by measuring the effects of the test compounds on calcium flux induced by 15 nM human NPY (Catalog No. 4287). A concentration-response curve was generated to calculate IC_50_ values.

### 2.7. Statistical Analyses

Data are presented as mean ± standard error. Student's or Aspin–Welch's *t*-test was performed between vehicle-treated mice and CRF-injected mice. For multiple-group comparison, Dunnett test was used. Data were analyzed using StatLight 2000 (Yukms, Tokyo, Japan). In addition, *P* < 0.05 was considered significant.

## 3. Results

### 3.1. Delayed Gastric Emptying Was Induced by CRF but Ameliorated by BGH

Drug administration and gastric emptying measurement were performed, as shown in Figure 1(a). CRF ICV administration significantly decreased the gastric emptying rates of solid test meal at 0.3 and 1.0 *μ*g/mouse (Figure 1(b) and 1(c)). However, oral BGH administration (1.0 g/kg) significantly ameliorated the delayed gastric emptying induced by CRF at 0.3 *μ*g/mouse as shown in Figure 1(d).

### 3.2. BGH Enhanced Gastric Emptying Was in Normal Mice

Drug administration and gastric emptying measurement were performed, as shown in Figure 2(a). Oral BGH administration (0.5 and 1.0 g/kg) enhanced gastric emptying significantly at 1 hour (Figure 2(b)) and slightly at 2 hours (Figure 2(c)) after the test meal administration.

### 3.3. *In Vitro* Functional Assay to Assess Receptor and Enzyme Antagonistic Activities of BGH Components

**Figure 3:**
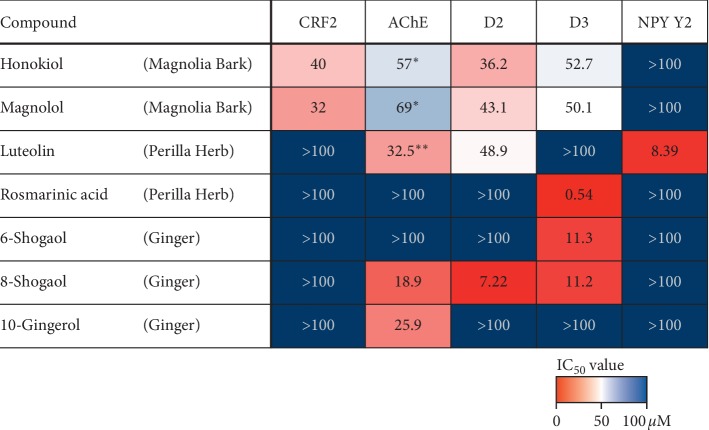
Antagonistic activities against each target are shown as IC_50_ values (*μ*M) in the table and colored according to a color scale. CRF2, corticotropin-releasing factor type-2 receptor; AChE, acetylcholinesterase; D2/D3, dopamine D2/D3 receptor; NPY Y2, neuropeptide Y Y2 receptor. ^*∗*^ and ^*∗∗*^: reported values from the previous studies [23, 24].

Antagonistic activities of BGH components were evaluated against human CRF2 receptor, AChE, dopamine D2 and D3 receptors, and NPY Y2 receptor. The IC_50_ values against each target are shown in Figure 3 and colored according to a color scale. Components showing strong inhibitory activities, smaller IC_50_ values, are colored red. For instance, 8-shogaol derived from ginger, rosmarinic acid, and luteolin from Perilla Herb showed strong antagonistic effects against dopamine D2/D3 and NPY Y2 receptor among tested compounds (IC_50_ = 7.22, 0.54 and 8.39 *μ*M, respectively).

## 4. Discussion

**Figure 4:**
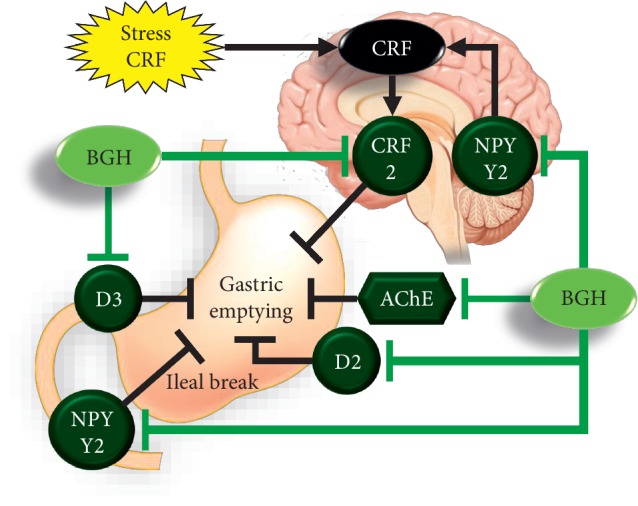
Hypothesis to explain the effect of bukuryoingohangekobokuto (BGH) on stress hormone–induced delayed gastric emptying. Intracerebroventricular administration of the stress hormone CRF delays gastric emptying via CRF2 receptor. BGH may exert its restorative effect via CRF2 antagonism. Moreover, BGH may enhance gastric emptying via increasing gastric motility by acetylcholine production through D2 receptor and AChE antagonism and via relaxing the pyloric sphincter by D3 receptor antagonism. NPY Y2 antagonism may contribute to the suppression of the CRF pathway and ileal break. CRF, corticotropin-releasing factor; CRF2, CRF type-2 receptor; D2/D3, dopamine D2/D3 receptor; AChE, acetylcholinesterase; NPY Y2, neuropeptide Y Y2 receptor. The image depicted in this figure is our own.

The present study exhibited the following: (1) the Kampo medicine BGH ameliorated delayed gastric emptying induced by the stress hormone CRF via ICV injection, (2) BGH also enhanced gastric emptying in normal mice, (3) several components of BGH showed antagonistic effects against CRF2 receptor, dopamine D2 and D3 receptors, NPY Y2 receptors, and AChE that may be attributable to BGH's improving effects on gastric emptying (Figure 4).

CRF plays a significant role in the central nervous system and is secreted as an adaptive response to stress. CRF pathways strongly influence anxiety- and stress-related behaviors [25], but CRF antagonists suppress these effects [26]. The involvement of CRF is also confirmed by CRF-overexpressing or CRF-knockout mice [27, 28]. In rodents, centrally administered CRF induces stress-like behaviors, such as increased depression, decreased rearing activity, suppression of food intake [29, 30], and delayed gastric emptying, which is associated with the activation of a CRF2 receptor subtype [31]. In the present study, the ICV administration of CRF significantly induced delay in gastric emptying, but the delay was relieved by BGH, indicating that BGH might have blocked the CRF-CRF2 pathway. One of the possible reasons is that Magnolia Bark–derived magnolol and honokiol contributed to the effects, considering that they possess CRF2 receptor antagonistic activities, which we reported for the first time and reportedly capable of traversing the blood-brain barrier [32, 33].

BGH not only recovered the delayed gastric emptying of CRF-administered mice but also accelerated the gastric emptying in normal mice in this study. Dopamine D2 receptor antagonists and AChE inhibitors, such as metoclopramide, domperidone, itopride, and acotiamide, are well-known gastrokinetic agents. Considering that some of the BGH components (e.g., 8-shogaol, honokiol, magnolol, and luteolin) can inhibit dopamine D2 receptor, which we reported for the first time, they might have contributed to the gastrokinetic effects. Among the four compounds, 8-shogaol showed the strongest inhibitory activities against dopamine D2 receptor, and it may act only peripherally because 8-shogaol was not detected in the brain after the oral administration of ginger extract [34]. Some compounds also inhibited AChE, although their contribution to the effect of BGH might be limited, given that they only possessed moderate activities.

Dopamine D3 receptor activation delays gastric emptying by impairing pyloric relaxation, and the D3 receptor antagonist partially reversed the effect of dopamine on gastric emptying [35]. Therefore, we investigated the effect of BGH components on D3 receptor and found that rosmarinic acid derived from Perilla Herb and 6-shogaol, 8-shogaol derived from ginger possess antagonistic activities that may contribute to the effect of BGH. Although computational approaches revealed the possibility of rosmarinic acid as a ligand for D3 receptor [36], this study is the first to show their antagonistic effects. Rosmarinic acid may exert its effect only in the peripheral organs because its blood-brain barrier permeability is extremely low [37] and detected only in plasma after its oral administration.

Peptide YY (PYY) is one of the major anorexigenic, gastrointestinal, neuroendocrine peptides mediated by NPY Y2 receptor [38], and it inhibits various gastrointestinal functions, including gastric acid secretion, gastric emptying, and intestinal transit [39], commonly known as the “ileal break.” Peripheral and central injection of PYY inhibits gastric emptying in rodents [40, 41], and PYY−/− mice reportedly have faster upper gastrointestinal transit than wild-type mice under normal conditions [42] and during acute stress [43]. Therefore, we investigated the effect of BGH components on NPY Y2 receptor. To the best of our knowledge, we found for the first time that luteolin derived from Perilla Herb possesses antagonistic activities, which may contribute to the restorative effect on delayed gastric emptying. NPY Y2 receptor expressed in the central nervous system regulates neuropsychiatric mood conditions [44, 45]. Deletion of Y2 receptors causes a reduction of CRF mRNA expression [46], indicating the possibility that reduced anxiolytic-like properties in the Y2−/− mice are partly attributable to the inhibition of the CRF pathways. Therefore, BGH may exert ameliorative effects to the psychological symptoms because luteolin is reportedly capable of crossing blood-brain barrier [47].

Adrenergic α2 antagonism can relieve delayed gastric emptying induced by the urocortin 1–CRF2 pathway [48]. Considering that 6-shogaol, 8-shogaol, and 10-gingerol derived from ginger and synephrine derived from *Citrus unshiu* peel reportedly showed adrenergic α2 antagonistic activities [49], these compounds might be involved in the restorative effect of BGH in CRF-induced delayed gastric emptying model, which was observed in this study. Magnolol, honokiol [50], and ginger extract [51] can accelerate gastric emptying, although their precise mechanisms remain unclear. Moreover, BGH components may possibly act on other gastric motility‐related receptors, such as muscarinic type 3, serotonin type 3/4, cholecystokinin, ghrelin, motilin, and GLP-1 receptors; however, further studies are needed to fully understand the action mechanisms of BGH.

## 5. Conclusions

BGH improves gastric emptying probably via various targets, mostly dopamine D2/D3 and NPY Y2 receptor antagonism, in mice. Therefore, BGH might be a useful therapy for patients that suffer both gastrointestinal and psychological symptoms and may contribute to the improvement of quality of life and the prevention of polypharmacy in the elderly.

## Data Availability

All data generated or analyzed during this study are included in this published article.

## Abbreviations

AChE: Acetylcholinesterase
BGH: Bukuryoingohangekobokuto
CRF: Corticotropin-releasing factor
CRF2: CRF type-2 receptor
ICV: Intracerebroventricular
NPY: Neuropeptide Y.
